# No extraction? No problem. Direct to PCR processing of tongue swabs for diagnosis of tuberculosis disease as an alternative to sputum collection

**DOI:** 10.1128/spectrum.03107-23

**Published:** 2023-12-08

**Authors:** Charlotte L. Ahls, Duncan Emsweller, Seth J. Helfers, Xin Niu, Douglas Wilson, Leah R. Padgett, Paul K. Drain

**Affiliations:** 1 Quantigen LLC, Fishers, Indiana, USA; 2 Department of Global Health, University of Washington, Seattle, Washington, USA; 3 Umkhuseli Innovation and Research Management, Pietermaritzburg, South Africa; 4 Department of Internal Medicine, Harry Gwala Regional Hospital, University of KwaZulu-Natal, Pietermaritzburg, South Africa; 5 Department of Medicine, University of Washington, Seattle, Washington, USA; Quest Diagnostics, San Juan Capistrano, California, USA

**Keywords:** *Mycobacterium tuberculosis*, tongue swab, qPCR, direct lysis

## Abstract

Sputum collection and testing for tuberculosis (TB) have been problematic due to the potential for aerosolization, difficulty in generating a quality sample, and complex DNA extraction methods. Tongue swabs are inexpensive, minimally invasive, and a promising alternative to sputum collection. We investigated the diagnostic accuracy of tongue swabs for TB diagnosis using the Truenat MTB Plus assay from Molbio Diagnostics with a direct to PCR processing method. Four tongue swabs were collected per participant using two nylon-flocked swabs and two spun polyester swabs. Following tongue swab sample collection, participants also provided two sputum samples, which were tested by Cepheid Xpert MTB/RIF Ultra or culture. Of the 81 participants enrolled, 24 (30%) were positive for TB disease by sputum Ultra. Using the Truenat MTB Plus test, tongue swabs had 54% (52/96) sensitivity and 99% (218/220) specificity compared to sputum Ultra. Crude lysate was also tested using an in-house qPCR assay, which allowed for increased sample input. Using this method, tongue swabs had 70% (67/96) sensitivity and 94% (216/224) specificity. *Mycobacterium tuberculosis* (MTB) sample quantification using digital PCR yielded between 20 copies (minimum) and 34,000 copies (maximum) of MTB per swab. In addition, serially collected tongue swabs resulted in similar levels of detected MTB, and spun polyester swabs performed equivalently to nylon-flocked swabs. Overall, this study demonstrates that tongue swab samples are compatible with the Truenat MTB testing platform and that a direct to PCR method is a viable diagnostic solution.

**IMPORTANCE:**

Tuberculosis (TB) remains one of the world’s leading infectious disease killers, despite available treatments. Although highly sensitive molecular diagnostics are available, expensive equipment and poor infrastructure have hindered their implementation in low-resource settings. Furthermore, the collection of sputum poses challenges as it is difficult for patients to produce and creates dangerous aerosols. This manuscript explores tongue swabs as a promising alternative to sputum collection. While previous studies have explored the sensitivity of tongue swabs as compared to sputum, existing literature has not addressed the need to standardize and simplify laboratory processing for easy implementation in high TB burden areas. This manuscript provides the first evidence that detection of TB from a tongue swab is possible without the use of DNA extraction or purification steps. The data provided in this manuscript will improve the collection and testing of tongue swabs for the diagnosis of TB disease.

## INTRODUCTION

There is an urgent need to transform the landscape of tuberculosis (TB) diagnostics, which claimed more than 1.3 million lives in 2021 (1, 2). Although new, highly sensitive molecular diagnostic tests have been developed, their implementation in high-burden areas is often not feasible due to their high cost and need for expensive laboratory infrastructure (3 – 5). These devices are further limited by their inability to detect TB in children and HIV-coinfected patients since these individuals often have difficulty producing the quality sputum samples required for diagnosis (6 – 8).

Detecting *Mycobacterium tuberculosis* (MTB) from sputum samples is problematic as it has a high potential for creating aerosols. The consistency of these samples requires both chemicals and extraction equipment for processing, adding cost and complexity to TB testing. Due to these factors, alternative sample types, such as saliva, urine, and blood, have been investigated. These alternative sample types have been less sensitive to detecting MTB compared to sputum sampling (9). In contrast, many studies have shown that MTB deposited on the tongue can be detected by PCR (10 – 13). Comparatively, tongue swabs achieved 92.8% sensitivity relative to sputum Xpert MTB/RIF Ultra (Ultra) when two tongue swabs were collected on consecutive days and tested using a PCR-based platform (14). Additional studies using Ultra to test TB tongue swabs have seen highly variable results ranging between 22 and 77.8% sensitivity (15 – 17), highlighting a need to optimize and standardize tongue swab processing for TB diagnostics.

In this study, we examined the diagnostic accuracy of tongue swab samples, determined the bacterial load collected via a single tongue swab, and evaluated serially collected tongue swabs using two swab types. Tongue swab samples were evaluated using the Molbio Truenat MTB Plus test, a World Health Organization (WHO)-approved TB diagnostic used with sputum samples (18, 19). This near-point-of-care (POC) test utilizes an automated extraction procedure (Trueprep) followed by a chip-based PCR to detect MTB DNA. This extraction requires a separate instrument, an extraction cartridge, and additional reagents, which adds cost and time. Tongue swabs, however, do not require the same intense chemical processing and extraction procedures as sputum. Therefore, we sought to examine tongue swab samples using an extraction-free process, defined as the omission of any DNA extraction or purification steps to separate nucleic acids from the sample. We determined that a simplified heat lysis in lieu of an extraction was sufficient for detecting MTB. In addition, we used digital PCR (dPCR) to determine the number of genomic copies of MTB present on a clinical tongue swab. Considering diagnostic accuracy is dependent on optimal sample collection, we also assessed the performance of two different swab types, a nylon-flocked swab and a spun polyester swab, to determine the optimal material for maximum recovery of MTB. The course of these studies establishes the compatibility of tongue swab samples with the Molbio Truenat MTB Plus test and provides the first evidence that an extraction-free method may be feasible for clinical testing.

## MATERIALS AND METHODS

### Study site and population

Adults (aged ≥16 years) at high risk for TB diagnosis due to TB-related symptoms, a positive sputum Ultra TB test, or HIV infection were consecutively enrolled into the prospective PROVE-TB cohort at Harry Gwala Hospital and affiliated clinics in Pietermaritzburg, South Africa, between October 2019 and February 2021. Persons who had received TB treatment for more than 24 hours were excluded. Clinical, laboratory, and demographic data were collected from participants and clinical charts. Sputum, urine, tongue swabs, and blood samples were collected and transported to the on-site laboratory for processing and analysis. Study data were collected and managed using REDCap electronic data capture tools (20) hosted at the Institute of Translational Health Sciences. All participants provided written informed consent. This study was approved by the ethical committees of the University of KwaZulu-Natal (BREC #BE475/18) and the University of Washington (UW). The protocol was submitted to the institutional review board of UW, who made a determination on non-engagement for this study.

### Sample collection

**FIG 1:**
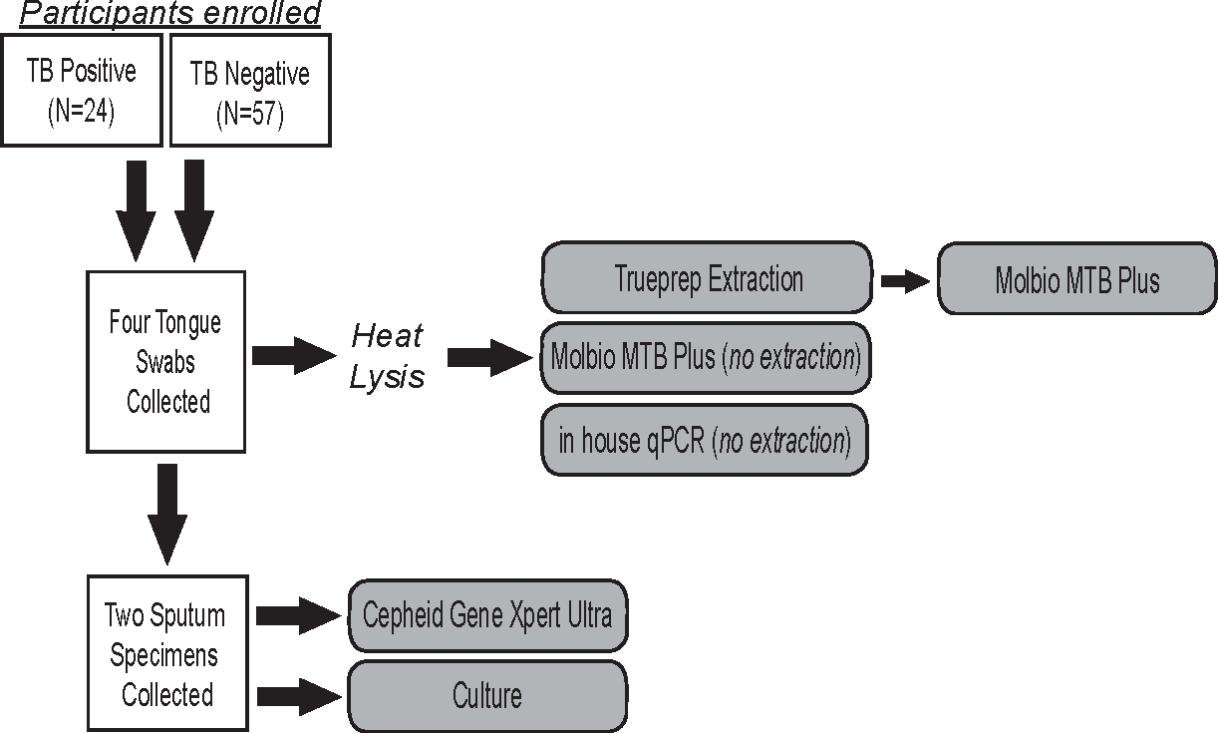
Workflow of sample collection and laboratory testing.

All tongue swabs were collected before two expectorated sputum specimens (Fig. 1). Subjects were instructed not to eat or drink for at least 30 minutes prior to swabbing. Swabbing was done on the tongue dorsum, as described in Laubeka et al. (14). In brief, the front two-thirds of the tongue dorsum were swabbed for 15 seconds while rolling the swab up and down and side to side and applying enough pressure to gently bend the swab. Four swabs were collected from each patient: two were collected using a spun polyester swab (SteriPack, #60567RevB) and two were collected using a nylon-flocked swab (ASP Medical, #8202-3). Swabs were collected in alternating order: even participant IDs starting with the SteriPack swab and odd participant IDs starting with the ASP swab. The 3-inch (72 mM) SteriPack swabs were collected into 15 mL falcon tubes (ThermoFisher, #339650), while the ASP swabs, which featured a 30-mM breakpoint, were collected into 2 mL screwcap microcentrifuge tubes (Fisher, #02-682-558). All swabs were collected dry, without buffer, and transported at 4°C. Swabs were stored at −80°C until processing.

### Sputum testing and culture

Expectorated sputum samples were tested with Cepheid GeneXpert MTB/RIF Ultra and mycobacterial culture (National Health Laboratory System, South Africa). A TB case was defined as a participant with a positive result from either sputum Ultra or sputum TB culture (reference testing). A sample was considered negative if both Ultra and culture resulted negative, or negative by one and the other was not done/resulted. Ultra also provides a semi-quantitative result: high, medium, low, very low, and trace (all considered positives) and negative.

### Swab processing and detection

Swabs collected in 15 mL falcon tubes were transferred to 2 mL microcentrifuge tubes using sterile forceps. A 900-µL of Tris-EDTA buffer (TE) was added to elute each swab. Swabs were vortexed for 30 seconds and then heated for 30 minutes at 95°C to create the lysate (Fig. 1). In addition, 400 µL of lysate [modified from the 500 µL designated in the instructions for use (IFU)] was treated with the Trueprep AUTO MTB Sample Pre-treatment Pack (Molbio, #60204AS20) and extracted using the Trueprep AUTO v2 Universal Cartridge Based Sample Prep Kit (Molbio, #60207AR50). Moreover, 6 µL of Trueprep eluate was added to the MTB Plus assay chip (Molbio, #601130050). Samples that failed during extraction or displayed no amplification of the internal positive control (IPC) were not repeated, as there was not enough volume for repeat extractions from the same sample.

In addition, 6 µL of unextracted lysate was added directly to the MTB Plus assay chip. If an error occurred, the sample was re-run per the IFU on a new MTB Plus chip, and the result of the second chip was used for analysis. For all positive samples, the MTB Plus assay provides a target cycle threshold (CT) value and a semi-quantitative result: high, medium, low, and very low.

Lastly, 20 µL of lysate (unextracted) was tested using an in-house qPCR for MTB detection. The qPCR assay targeted the multicopy insertion elements IS6110 and IS1081 (Table S1). The final mix included 25 µL of TaqMan Gene Expression Master Mix (ThermoFisher, #4369016), 2.5 µL of 20× assay, 2.5 µL of nuclease-free H20, and 20 µL of lysate to bring the final reaction volume to 50 µL. qPCR was run using the QuantStudio 7 Flex instrument (ThermoFisher). A threshold of 0.04 was applied to all targets to standardize CT values across all qPCR plates. If only one MTB target was amplified with a CT ≥38, this sample was deemed inconclusive and repeated. If the sample was amplified again upon resting, the sample was considered positive. If no amplification was observed during the retest, the sample was considered negative.

**TABLE 1:** Tongue swab sensitivity and specificity relative to sputum Ultra and culture

	Tongue swab processing and detection method		
Analysis (Ultra reference standard)	Trueprep extraction with MTB Plus (*2.2% of sample tested*)	Direct to PCR with MTB Plus (*0.7% of sample tested*)	Direct to PCR with in-house qPCR (*2.2% of sample tested*)
Percent sensitivity (95% CI), n/N	51 (41–61), 49/96	54 (44–64), 52/96	70 (60–78), 67/96
Percent specificity (95% CI), n/N	99 (97–100), 209/211^*a*^	99 (97–100), 218/220^*b*^	94 (82–90), 216/224
Analysis (culture reference standard)			
Percent sensitivity (95% CI), n/N	73 (60–83), 41/56	79 (66–87), 44/56	98 (91–99), 55/56
Percent Specificity (95% CI), n/N	99 (97–100), 208/210^*c*^	99 (97–100), 214/216^*d*^	95 (91–97 81-90), 208/220

^*a*^ Thirteen samples were excluded due to extraction and/or PCR errors.

^*b*^ Four samples were excluded due to PCR errors.

^*c*^ Ten samples were excluded due to extraction/PCR errors.

^*d*^ Four samples were excluded due to PCR error.

### Digital PCR

Trueprep eluate that tested positive for MTB on the Molbio MTB Plus assay was quantified by dPCR. Protein antigen b (PAB) was targeted for quantification. PAB primers and probes were designed in-house (Table S2). The final reaction mix included 1.8 µL of Absolute Q DNA Digital PCR Master Mix (ThermoFisher, #A52490), 0.45 µL of 20× assay, 2.75 µL of nuclease-free water, and 6 µL of Trueprep eluate to bring the final reaction volume to 9 µL. dPCR was run on the QuantStudio Absolute Q Digital PCR System (ThermoFisher). A standard threshold for each plate was set equal to the automatically determined threshold for the positive control.

**TABLE 2:** MTB detected on tongue swabs as compared to the paired sputum ultra semi-quantitative result

	Ultra semi-quantitative result			
Number of swabs detected by each method	High	Med	Low	Trace
Trueprep extraction with MTB Plus	16/20 (80%)	5/16 (31%)	20/40 (50%)	0/8 (0%)
Direct to PCR with MTB Plus	16/20 (80%)	6/16 (38%)	22/40 (55%)	0/8 (0%)
Direct to PCR with in-house qPCR	17/20 (85%)	10/16 (63%)	31/40 (78%)	0/8 (0%)

### Statistical analysis

All tongue swabs were analyzed individually for the purpose of this study. Positive results, as determined by the specific molecular test, indicate a positive on a singular tongue swab. All statistical analysis was done using Graph Prism (v9.4.0). Sputum Ultra and sputum culture were used as the reference standards to calculate sensitivity and specificity. 95% confidence intervals (95% CI) were calculated using the Wilson-Brown method. An unpaired *t*-test was used to analyze differences in CT values between HIV-positive and HIV-negative participants. Pearson’s correlation matrix was used to examine the impact of collection order on CT values. Pearson’s correlation matrix was also used to analyze differences in detection between ASP and SteriPack swabs. A CT value of 40.0 was used in the correlation matrix to represent swabs where MTB was not detected. A 95% CI and *P* value of 0.05 were used for significance for all testing groups.

## RESULTS

### Cohort analysis

**FIG 2:**
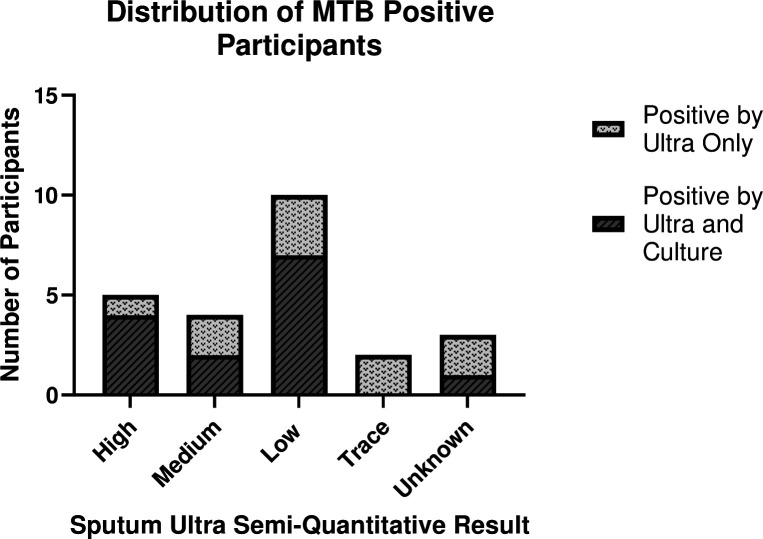
Diagram of MTB-positive participants by two reference standards (i) Ultra and (ii) culture. Participants whose Ultra semi-quantitative results were not provided are categorized as Unknown.

A total of 81 participants were enrolled in this study: 47 males and 34 females. Ultra results were available for 80 of the 81 participants. Moreover, 24 participants (29.6%) were confirmed TB positive by sputum Ultra (5 high, 4 medium, 10 low, 2 trace, and 3 participants where a semi-quantitative result was unavailable), and 14 of these participants were also confirmed positive by culture (Fig. 2). Six participants positive by Ultra were culture-negative. All culture-positives were also positive by Ultra. Excluding contaminated samples and unavailable culture results (*n* = 4), the positive percent agreement between Ultra and culture was 70% (95% CI: 48–86%). Among the 24 participants positive by Ultra, 15 were also positive for HIV.

### Tongue swab performance

To determine the optimal processing method for TB tongue swabs, the samples were first eluted in 900 µL of TE, vortexed, and heat-inactivated at 95°C for 30 minutes. The diagnostic accuracy was then assessed across three methods: (i) lysate extracted with Trueprep and tested with MTB Plus, (ii) lysate tested directly with MTB Plus, and (iii) lysate tested directly using an in-house qPCR. Trueprep extracted swabs tested with MTB Plus (*n* = 322) had 51% (95% CI: 41–61%) sensitivity with 99% (95% CI: 97–100%) specificity compared to sputum Ultra (Table 1) and 73% sensitivity (95% CI: 60–83%) with 99% specificity (95% CI: 97–100%) compared to sputum culture. Three samples (1%) had failures during extraction, and 10 samples (3%) failed during qPCR due to no amplification of IPC.

Considering the Truenat extraction increases cost and time to result, we sought to determine if the extraction step could be bypassed and tongue swab lysate could be added directly to the Molbio PCR chip for testing. Tongue swabs tested using this direct to PCR method with the MTB Plus assay (*n* = 324) had 54% (95% CI: 44–64%) sensitivity and 99% (95% CI: 97–100%) specificity compared to sputum Ultra and 79% sensitivity (95% CI: 60–83%) with 99% specificity (95% CI: 97–100%) compared to sputum culture. In addition, 8% (26/324) of samples displayed errors during PCR, which were repeated per the Molbio Truenat protocol. Of the samples retested, only four had errors upon the second test, resulting in an overall 1% (4/324) error rate.

The direct to Molbio PCR method provided the first evidence that extraction is not required to detect MTB from a tongue swab. However, this method limits the tested sample volume to 6 µL or 0.7% of the original sample. To increase the sample volume tested from 6 µL to 20 µL, the tongue swabs were also tested using a direct to PCR method with an in-house qPCR assay (*n* = 324). This method yielded 70% (95% CI: 60–78%) sensitivity and 94% (95% CI: 90–96%) specificity compared to sputum Ultra and 98% sensitivity (95% CI: 91–99%) with 95% specificity (95% CI: 91–97%) compared to sputum culture. Moreover, 7% (22/324) of samples tested inconclusive and were retested. When comparing these results to the sputum Ultra semi-quantitative result, this method successfully detected 85% (17/20) of the Ultra high positives, 63% (10/16) of the medium positives, 78% (31/40) of the low positives, and none of the trace positives (0/8) (Table 2).

The analysis of tongue swab performance was determined using four swabs per participant. However, if using only the first collected swab to analyze qPCR concordance to sputum Ultra, sensitivity was 71% (95% CI: 51–85%) and specificity was 96% (95% CI: 88–99%), which closely aligns with the total swab analysis. Of the 19 participants with positive tongue swabs, 17 had a positive on the first tongue swab collected.

Of the tongue swabs that tested positive for MTB by qPCR, mean CTs were significantly higher in HIV-positive individuals (mean CT = 34.67) compared to HIV-negative individuals (mean CT = 30.60) (*P* value < 0.0001).

### Quantitation of bacterial load via tongue swab

**FIG 3:**
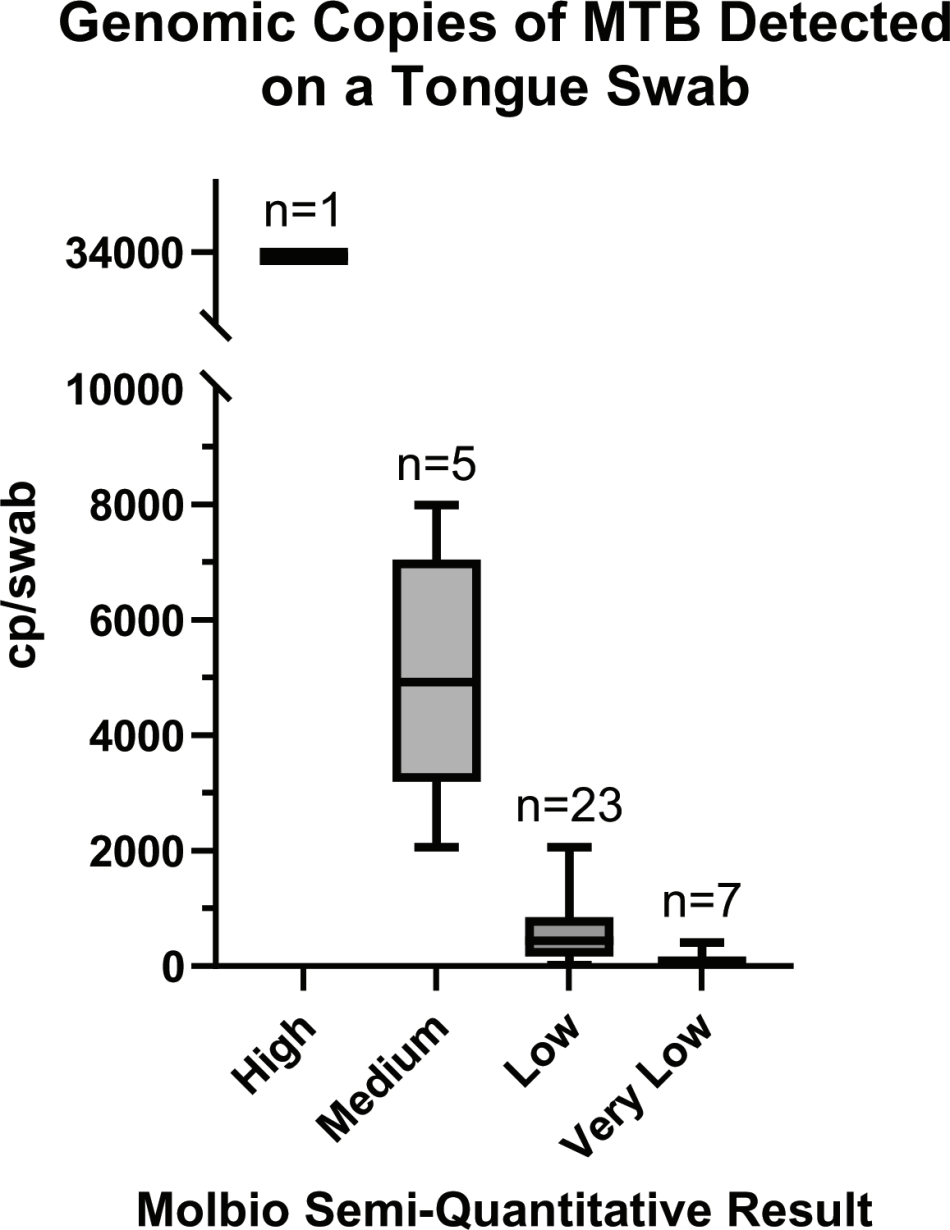
Genomic copies detected per tongue swab (*N* = 36) for samples identified as TB-positive by the Truenat MTB Plus assay.

Adapting tongue swab samples for TB diagnostics requires a thorough understanding of bacterial loads, as this information is critical for optimizing processing workflows and downstream testing platforms. Therefore, we performed dPCR to determine the number of bacteria collected on a clinical TB tongue swab. Quantifiable results were obtained for 36 MTB-positive tongue swab samples using the Trueprep eluate analyzed by dPCR. The single “high positive” tongue swab identified by the MTB Plus assay had 33,900 MTB copies/swab (*n* = 1). Swabs identified as “medium positive” had an average of 5,080 copies/swab (*n* = 5). “Low positives” had an average of 600 copies/swab (*n* = 23), and “very low positives” had an average of 94 copies/swab (*n* = 7) (Fig. 3; Table S3). The lowest quantifiable amount of MTB detected by dPCR was 20 copies/swab. These data establish that tongue swabs are sufficient for collecting a wide range of bacterial loads.

### Analysis of collection order

**FIG 4:**
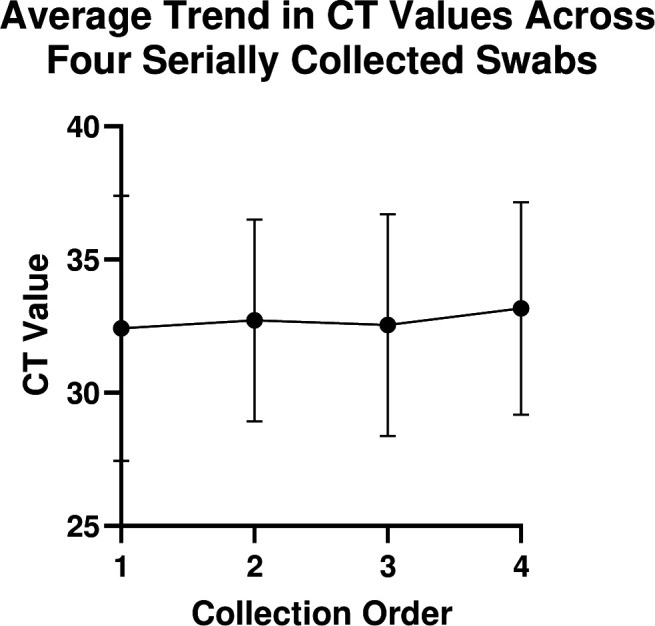
CT values (using in-house qPCR) from four serially collected swabs for 18 TB-positive participants were averaged and plotted in order of collection.

Evaluating tongue swab performance across multiple TB diagnostic workflows is challenging due to the inherent variability of sample collection. Therefore, we sought to determine if four serially collected tongue swabs performed the same within a single assay. Of the 24 TB-positive participants, 14 (54%) had positive qPCR results on all four tongue swabs, four (17%) had 2–3 swabs positive by qPCR, one participant had a singular positive swab (4%), and five (21%) had no positive swabs (Table S4). Participants who had fewer than four positive swabs also had very low bacterial loads detected (mean CT = 37.4, ±1.44). CT values obtained from the in-house qPCR assay were assessed to determine trends across collection order for the 18 positive participants with more than one qPCR-positive swab. No significant correlation was detected between collection order and CT value (*P* value = 0.1856) (Fig. 4). These data indicate that serially collected tongue swabs result in similar amounts of collected bacteria and, thus, could be used for comparator studies.

### Analysis of swab type

**FIG 5:**
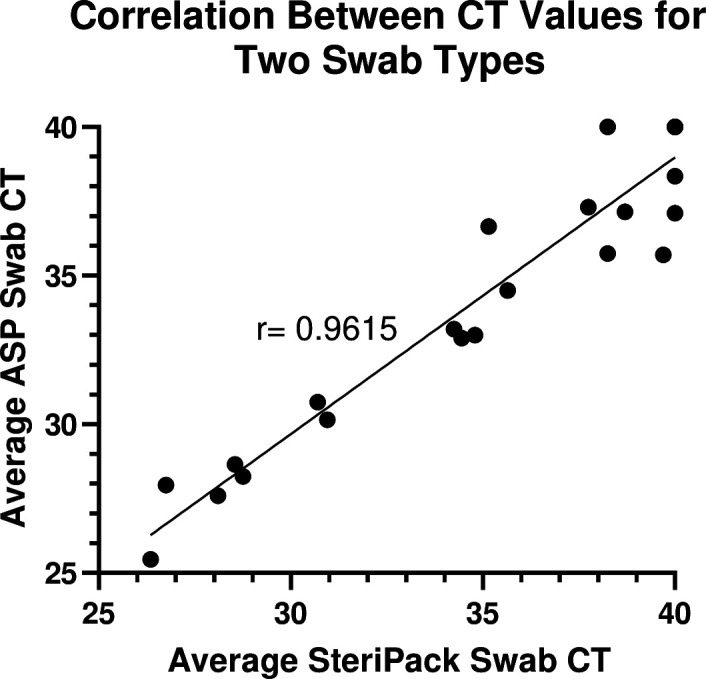
XY correlation plot displaying average CT values for each swab type for 24 TB-positive participants. Significant correlation (Pearson *r* = 0.96, *P* value < 0.0001).

Swab type could be a key determinant in tongue swab sample collection as there are varying bud sizes, stem lengths, and materials. In this study, we evaluated the performance of a nylon-flocked swab (ASP) and a spun polyester swab (SteriPack). Of the 96 swabs collected from participants with confirmed TB (48 ASP and 48 SteriPack), 36 ASP swabs (75%) and 31 SteriPack swabs (65%) were positive by qPCR (Table S4). A strong correlation was observed between CT values from the SteriPack and ASP swabs (Pearson *r* = 0.96, *P* value < 0.0001) (Fig. 5) indicating equivalent performance between swab types.

## DISCUSSION

In this study, we determined the compatibility of tongue swab samples using an extraction-free process with a WHO-approved, PCR-based test for detecting MTB. Of the three methods assessed in this study, tongue swabs processed with direct lysis and tested using an in-house qPCR provided the highest sensitivity with regard to sputum Ultra and sputum culture. Altogether, these data show that tongue swabs are compatible with the Molbio PCR platform and that direct lysis is a feasible diagnostic workflow. Increasing the sample input from 6 µL Laubeka (Truenat MTB Plus) to 20 µL (in-house qPCR) resulted in a sensitivity increase from 54% to 72%. This highlights the need for larger sample inputs to achieve higher concordance with sputum diagnostics.

Previous studies reported 71–94% tongue swab sensitivity as compared to sputum Ultra testing (10, 11, 14), all of which utilized DNA extraction and/or ethanol precipitation protocols to concentrate and purify the sample. This study is the first to demonstrate that the same level of sensitivity can be achieved using heat only in combination with a highly sensitive, inhibitor-tolerant qPCR. Although thermal lysis has been shown to be effective at inactivating TB (21, 22), it is also preferable as it does not require expensive equipment or chemicals that could interfere with downstream molecular detection. Minimizing the number of processing steps and skills required to conduct these tests is crucial for adapting tongue swabs as a diagnostic tool, particularly in a POC setting (23).

To build upon the results shown here, future studies should explore methods for increasing lysis efficiency without adding expensive equipment that may be unavailable in low- and middle-income countries. Avenues of interest include mechanical bead beating via a simple laboratory vortex mixer, shaking by hand, or smaller, battery-operated bead beaters that could be integrated into a POC test (24, 25). Improving lysis efficiency would also aid in accurately quantifying the amount of MTB found on a tongue swab. When comparing the semi-quantitative MTB Plus result to the semi-quantitative sputum Ultra result, tongue swabs were most often classified as having fewer bacterial loads than their sputum counterparts (Table S3). However, more efficient lysis methods should help to bridge this gap.

Other methods for improving sensitivity should focus on optimizing the collection and storage of tongue swab samples to maximize the pickup and release of MTB. The ASP swab did result in a 10% increase in detection, which could be due to the larger swab head and increased surface area for collecting material, particularly in participants with low bacterial loads. Despite the smaller size of the swab head, the strong correlation between CT values suggests that the polyester swab, which was validated for use as a nasal collection device for SARS-CoV-2 (26), could also be a suitable collection device for TB tongue swabs. The consistency of bacterial loads across four serially collected swabs suggests that neither swab type had the capacity to exhaustively sample available MTB; therefore, using swabs with a higher capacity for bacteria pickup could result in greater sensitivity.

Many areas with a high TB burden do not have access to advanced diagnostic tools due to a combination of economic barriers, lack of equipment, and poor training (2, 3). Although more methods need to be investigated to improve sensitivity, this study is the first to demonstrate the feasibility of bypassing extraction and using crude lysate directly on Molbio’s Truenat detection instrument. Reducing the burden of processing would accelerate the time to result while also reducing the cost per test. With further processing and lysis optimization, this methodology could broaden access to TB testing throughout endemic countries.

## SUPPLEMENTAL MATERIAL

Supplemental material spectrum.03107-23-s0001.pdf Tables S1 to S4.

ASM does not own the copyrights to Supplemental Material that may be linked to, or accessed through, an article. The authors have granted ASM a non-exclusive, world-wide license to publish the Supplemental Material files. Please contact the corresponding author directly for reuse.
