# Supplementary material for: No extraction? No problem. Direct to PCR processing of tongue swabs for diagnosis of tuberculosis disease as an alternative to sputum collection
Source: Microbiol Spectr. 2023 Dec 8;12(1):e03107-23. doi: 10.1128/spectrum.03107-23 (PMC10783077; doi:10.1128/spectrum.03107-23)
Supplement: Supplemental material — Tables S1 to S4. [file spectrum.03107-23-s0001.pdf]

SUPPLEMENTARY MATERIAL

Supplementary Table 1. Primer and probe sequences for MTB assay.

**Component**	**Sequence**	**Amplicon Length**
IS6110 Forward primer	AGCGCCGCTTCGGAC	
IS6110 Reverse primer	AGGCGTCGGTGACAAAGG	
IS6110 probe	/56-FAM/CGGCTGTGG/ZEN/GTAGCAGACCTCACC/3IABkFQ/	105bp
IS1081 Forward primer	CCTGCTGCACTCCATCTAC	
IS1081 Reverse primer	GGGAGTTTGTCGGTCAGAG	90bp
IS1081 probe	5HEX/CGCCGAATC/ZEN/AGTTGTTGCCCAAT/3IABkFQ	
RNaseP Forward primer	AGATTTGGACCTGCGAGCG	
RnaseP Reverse primer	GAGCGGCTGTCTCCACAAGT	65bp
RnaseP probe	/5Cy5/TTCTGACCT/TAO/GAAGGCTCTGCGCG/3IAbRQSp/	

Supplementary Table 2. PAB Assay for dPCR

**Component**	**Sequence**	**Amplicon Length**
PAB Forward Primer	GTCCTCGCGAGTCTAGGCCA	241

PAB Reverse Primer	TCCGCTGCCAGTCGTCTTCC	
PAB probe	56-FAM/CGCGGAACG/ZEN/TGGGACCAATACCTG /3IABkFQ	

Supplementary Table 3. Sputum Ultra semi-quantitative result compared to tongue swabs extracted using Molbio Trueprep and quantified with dPCR. Samples where Ultra semi-quantitative results were not provided are indicated by N/A.

Serial ID	Swab Type	Sputum Ultra Result	Tongue Swab Testing on Molbio Eluate		
			CT	Semi Quantitative Result	Average copies/swab
82001063	SteriPack spun polyester	High	19.8	High	33943
82001047	SteriPack spun polyester	High	23.75	Medium	6106
82001054	ASP nylon flocked	High	22.4	Medium	7986
82001054	ASP nylon flocked	High	24.57	Medium	2064
82001054	SteriPack spun polyester	High	23.6	Medium	4328
82001063	ASP nylon flocked	High	22.67	Medium	4924

82001024	ASP nylon flocked	High	28.0	Low	317
82001024	ASP nylon flocked	High	29.0	Low	211
82001024	SteriPack spun polyester	High	28.1	Low	346
82001047	ASP nylon flocked	High	25.2	Low	2063
82001047	ASP nylon flocked	High	26.5	Low	847
82001047	SteriPack spun polyester	High	27.14	Low	501
82001054	SteriPack spun polyester	High	25.33	Low	1563
82001063	ASP nylon flocked	High	26	Low	830
82001063	SteriPack spun polyester	High	25.25	Low	1507
82001008	ASP nylon flocked	Low	26.6	Low	675
82001008	ASP nylon flocked	Low	26.29	Low	1041
82001008	SteriPack spun polyester	Low	26.67	Low	310
82001008	SteriPack spun polyester	Low	27.43	Low	443
82001060	ASP nylon flocked	Low	29.8	Low	77
82001068	ASP nylon flocked	Low	29.5	Low	59
82001067	ASP nylon flocked	Medium	29.8	Low	56
82001005	ASP nylon flocked	N/A	29.1	Low	153

82001005	SteriPack spun polyester	N/A	27.7	Low	174
82001005	SteriPack spun polyester	N/A	26.3	Low	19
82001037	ASP nylon flocked	N/A	25.17	Low	984
82001037	ASP nylon flocked	N/A	27	Low	559
82001037	SteriPack spun polyester	N/A	28.29	Low	252
82001037	SteriPack spun polyester	N/A	25.33	Low	811
82001024	SteriPack spun polyester	High	30.3	Very Low	39
82001011	ASP nylon flocked	Low	31.3	Very Low	95
82001011	SteriPack spun polyester	Low	32.3	Very Low	19
82001060	ASP nylon flocked	Low	32.8	Very Low	409
82001060	SteriPack spun polyester	Low	35.8	Very Low	19
82001068	SteriPack spun polyester	Low	33.0	Very Low	19
82001067	ASP nylon flocked	Medium	31.0	Very Low	58

Supplementary Table 4. Comparison of MTB detection between two swab types using direct lysis processing and qPCR detection.

**CT Values Arranged by Swab Type**				
**Serial ID**	**SteriPack CT**		**ASP CT**	
82000767	*No Amp*	*No Amp*	*No Amp*	*No Amp*
82000769	*No Amp*	*No Amp*	*No Amp*	*No Amp*
82000778	*No Amp*	*No Amp*	*No Amp*	*No Amp*
82001005	29.6	31.8	30.2	31.3
82001008	28.7	27.5	27.9	27.3
82001011	33.7	35.2	32.7	33.1
82001014	39.3	37.2	35.6	35.9
82001024	30.6	31.3	30.1	30.2
82001025	*No Amp*	*No Amp*	*No Amp*	*No Amp*
82001028	36.5	*No Amp*	*No Amp*	*No Amp*
82001037	29.5	28.0	28.2	28.3
82001047	27.0	30.1	27.8	29.5
82001052	38.7	*No Amp*	35.9	38.4
82001053	*No Amp*	*No Amp*	36.8	37.4
82001054	27.1	25.6	24.6	26.3
82001058	35.5	34.8	35.7	37.6
82001060	33.6	36.0	32.2	33.8
82001061	*No Amp*	*No Amp*	*No Amp*	*No Amp*

82001063	24.9	28.6	26.9	29.0
82001064	*No Amp*	*No Amp*	38.2	38.5
82001065	37.0	38.5	36.7	37.9
82001066	39.7	*No Amp*	36.6	34.8
82001067	33.6	34.9	33.5	32.9
82001068	35.2	36.1	34.3	34.7
**Total Detected**	**31**		**36**	
